# Comparison of methods to determine accurate dose calibrator activity measurements

**DOI:** 10.1186/1756-9966-27-14

**Published:** 2008-07-01

**Authors:** Lidia Strigari, Marcello Benassi, Pierino De Felice, Marco D'Andrea, Aldo Fazio, Sandro Nocentini, Annelisa d'Angelo, Alessia Ceccatelli

**Affiliations:** 1 Laboratory of Medical Physics and Expert Systems, National Cancer Institute Regina Elena, Rome, Italy; 2 ENEA – Istituto Nazionale di Metrologia delle Radiazioni Ionizzanti, C.R. Casaccia, P.O. Box 2400 – I-00100, Rome, Italy

## Abstract

**Background:**

In nuclear medicine, liquid radiopharmaceuticals for diagnostic or therapeutic purposes are administered to patients by using various types of syringes with different volumes. The activity of each "dose" must be carefully measured and documented prior to administration using an activity calibrator.

**Methods:**

Calibrator response is a function of the measurement geometry and, in particular, it depends on the syringe type and filling volume. To minimize the uncertainty associated with the measured activity of the syringe, it is necessary to calculate a calibration curve depending on filling volume for each syringe type. This curve can be obtained by fitting experimentally determined volume correction factors.

**Results:**

A theoretical evaluation of volume correction factors for syringes is reported for three different experimental methods. The aim is to determine the most accurate experimental method among those considered, by examining the expression of uncertainty for the correction factor. This theoretical analysis was then tested experimentally.

**Conclusion:**

The agreement between the experimental data obtained in the constant activity method and gravimetric method at constant specific activity and the small associated uncertainties show the accuracy of these two procedures; while the volumetric method at constant specific activity could lead to a wrong evaluation of the correction factors.

## Background

In nuclear medicine, liquid radiopharmaceuticals for diagnostic or therapeutic purposes are administered to patients by using various types of syringes at different filling volumes. The activity of radiopharmaceuticals delivered to patients must be accurately known both to fulfill the radioprotection requirements and to assure successful therapies and good quality imaging procedures. An upper accuracy limit of 10% [1] is generally recommended and prior to administration, the vial containing the specific radiopharmaceutical is put into a radionuclide calibrator for activity evaluation and then an aliquot of solution is withdrawn from the vial into a syringe for administration. Generally, the syringe is also measured using the calibrator to confirm the activity to be administered.

Because of the dependence of calibrator response on measurement geometry, it is necessary to have a specific calibration factor for each syringe type and filling volume.

At the National Cancer Institute, Regina Elena, Rome (IRE), a theoretical and experimental evaluation of volume correction factors and associated uncertainties was performed for three possible experimental procedures. The uncertainty associated to the correction factor can vary according to the experimental procedure followed to determine a volume calibration curve. Indeed, to calculate the overall uncertainty it is necessary to consider the various components of uncertainty that contribute to the final result.

The aim was to determine the most accurate method, so as to optimise diagnostic performance and patient radioprotection. This evaluation was carried out by comparing the three modes of uncertainty expressions and by verifying experimentally the theoretical issues. for measurements we used a radionuclide calibrator COMECER PET DOSE at the Regina Elena National Cancer Institute (IRE) and manufactured in Italy.

## Methods

We designed three different experimental procedures to obtain volume variations inside the syringe and to determine the volume correction factors: a) Constant activity method: addition of gravimetrically determined increasing quantities of inactive solution to a certain volume of radioactive solution already in the syringe; b) Volumetric method at a constant specific activity: addition of volumetrically determined quantities of radioactive solution withdrawn from the same master solution; c) Gravimetric method at a constant specific activity: similar to the method described at point b), but the aliquot of radioactive solution inside the syringe is determined gravimetrically. the analytical expression for the volume correction factor was obtained for each procedure and the associated overall uncertainty was expressed, in each case, as combined standard uncertainty by square-summing all the components. The first method allows to evaluate the pure geometric effect on the calibrator response but the second and the third methods could be hampered by a possible non-linearity of the calibrator response for varying activities of source. Assuming calibrator linearity in the considered range of activity, the three approaches described above seem to be equivalent for the evaluation of volume correction factors.

The three procedures were experimentally carried out by using four types of plastic syringes 10 ml (ICO), 5 ml, 2.5 ml, 1 ml (ARTSANA), filled with a 99 mTc solution. The characteristics and geometries of these syringes are reported in Table 1.

**Table 1:** Main characteristics of the considered types of syringes

**Description**	**Nominal** **capacity ** **(ml)**	**Graduation** **(ml)**	**Filling** **volume ** ** range (ml)**
ICO plastic syringe	10	0.2	2.0÷9.0 steps 2.0
ARTSANA plastic syringe	5	0.2	1.0÷5.0 steps 1.0
ARTSANA plastic syringe	2.5	0.1	0.5÷2.5 steps 0.5
ARTSANA plastic syringe	1	0.02	0.20÷1.00 steps 0.20

Gravimetric measurements were performed using an analytical precision balance OHAUS Analytical Plus AP 210 [2], with a sensitivity of 0.1 mg, a capacity of 210 g and a linearity of ± 0.2 mg.

Activity measurements were performed using a radionuclide calibrator Comecer PET DOSE at the IRE, constituted by an ionization chamber filled with Argon gas. Its main characteristics [3] and calibration factors with traceability to national primary standards for three specific radionuclides (^99m^Tc, ^111^In and ^131^I) have already been reported [4]. A linearity test of the calibrator response was performed by measuring a Tc-99m solution at different times. The readings were corrected for decay to the same reference time and showed a maximum percentage difference of 0.8 % in the activity range 3 GBq – 2 MBq. To check the calibrator stability, daily measurements were carried out using high-stability check sources of four radionuclides with different photon energy and chamber response: Co-57, Ba-133, Cs-137 and Co-60. The readings, corrected for decay, were in agreement within 1%.

An external removable support as source holder for each different syringe type was used to improve the position reproducibility of the syringe inside the calibrator. Air was sucked up in the needle to avoid the presence of radioactive solutions in the needle itself, before measuring the syringe in order to ensure good counting geometry.

In the constant activity method, volume variations inside the syringe were obtained by adding 9% physiological solution to an initial aliquot of Tc-99m eluate.

For each syringe, the initial volume of stock solution was chosen according to syringe size: about 0.2 ml for a 1 ml syringe, 0.5 ml for a 2.5 ml syringe, 1 ml for a 5 ml syringe, and 2 ml for a 10 ml syringe. The syringes containing the initial volumes of stock solution were put into the calibrator and ten consecutive activity measurements were carried out for each syringe. The volume of carrier solution added each time was about the same of the initial radioactive volume and the syringe was re-measured after each carrier solution addition. The same experimental protocol was carried out for volumetric and gravimetric methods at constant specific activity, but, in these cases, volume variations were obtained by adding successive amounts of the same stock solution. The syringe reference geometry is equal to 40% of the nominal volume.

### Theory

The volume correction factor, K_*i*_, is defined by the following relation:

(1) $$A_{i}^{'}={\text{K}}_{i}\cdot A_{i}$$

where

A_i_= true activity in the i^th ^geometry

${\text{A}}_{\text{i}}^{\text{'}}$ = measured activity in the i^th ^geometry

### a) Constant activity method

Since the activity is constant, in this method, the true activity in the i^th ^geometry coincides with the measured activity in a reference geometry for the syringe, A_0_':

(2) $${\text{A}}_{\text{i}}=A_{0}^{'}=A_{0}$$

where A_0 _is the true activity in the syringe reference geometry.

From relation (1) the following expression for the correction factor can be obtained:

(3) $$K_{i}=\frac{{\text{A}}_{\text{i}}^{\text{'}}}{{\text{A}}_{\text{0}}^{\text{'}}}$$

On the basis of equation (3), the expression of the relative combined standard uncertainty u_*i*_, associated to K_*i*_, can be written as follows:

(4) $$u_{i}=\sqrt{{\left(s_{R_{i}}\right)}^{2}+{\left(s_{R_{o}}\right)}^{2}}$$

Where $s_{R_{i}}$ and $s_{R_{o}}$ are the relative standard deviations due to measurement reproducibility in the i^th ^and reference geometry respectively.

### b) Volumetric method at constant specific activity

Since, the specific activity, A_c_, in this method, is constant, from (1) follows:

(5) $${\text{A}}_{\text{i}}^{'}=K_{i}\cdot {\text{A}}_{\text{i}}=K_{i}\cdot {\text{A}}_{\text{c}}\cdot V_{i}.$$

where A_i _and V_i _are, respectively, the true activity and the syringe filling volume in the i^th ^geometry.

In the syringe reference geometry, we have:

(6) $${\text{A}}_{\text{0}}^{\text{'}}={\text{A}}_{\text{0}}={\text{A}}_{\text{c}}\cdot V_{0}$$

where

${\text{A}}_{\text{0}}^{\text{'}}$ = measured activity in the reference geometry

A_0_= true activity in the reference geometry

V_0_= syringe filling volume in the reference geometry.

Combining expressions (5) and (6) we obtain:

(7) $$A_{c}=\frac{{\text{A}}_{\text{0}}^{\text{'}}}{{\text{V}}_{\text{0}}}$$

Consequently, the expression for ${\text{A}}_{\text{i}}^{\text{'}}$ becomes:

(8) $${\text{A}}_{\text{i}}^{\text{'}}=\frac{{\text{A}}_{\text{0}}^{\text{'}}}{{\text{V}}_{\text{0}}}{\text{V}}_{\text{i}}\cdot {\text{K}}_{\text{i}}$$

and the following expression for the correction factor K_i _is obtained :

(9) $$K_{i}=\frac{{\text{A}}_{\text{i}}^{\text{'}}}{{\text{A}}_{\text{0}}^{\text{'}}}\cdot \frac{{\text{V}}_{\text{o}}}{{\text{V}}_{\text{i}}}$$

From equation (9), the expression of the relative combined standard uncertainty u_*i*_, associated to K_*i*_, can be written as follows:

(10) $$u_{i}=\sqrt{{\left(s_{R_{i}}\right)}^{2}+{\left(s_{R_{o}}\right)}^{2}+{\left(s_{R_{V_{i}}}\right)}^{2}+{\left(s_{R_{V_{0}}}\right)}^{2}}$$

In addition to the components due to measurement reproducibility in the i^th ^and reference geometries, in equation (10) relative contributions for syringe volume reading, $s_{R_{V_{i}}}$ and $s_{R_{V_{o}}}$, are also present. Volume uncertainty components due to syringe calibration do not appear because they are the same for V_0 _and V_i _measurements and, consequently, cancel out.

### c) Gravimetric method at constant specific activity

This method is an analogue to the one described at point b), but with mass in place of volume. Expression (9) for the correction factor K_*i *_becomes:

(11) $$K_{i}=\frac{{\text{A}}_{\text{i}}^{\text{'}}}{{\text{A}}_{\text{0}}^{\text{'}}}\cdot \frac{m_{o}}{m_{i}}$$

where m_0 _and m_i _are the masses of radiopharmaceutical in the syringe, in the reference and i^th ^geometries respectively.

From equation (11), the expression of the relative combined standard uncertainty u_*i*_, associated to K_*i*_, can be written as follows:

(12) $$u_{i}=\sqrt{{\left(s_{R_{i}}\right)}^{2}+{\left(s_{R_{o}}\right)}^{2}+{\left(s_{R_{m_{i}}}\right)}^{2}+{\left(s_{R_{m_{0}}}\right)}^{2}}$$

Mass uncertainty components due to balance calibration are the same for m_0 _and m_i _measurements and, consequently, cancel out.

## Results

Observing the mathematical expressions (4), (10) and (12) obtained for the relative combined standard uncertainty on K_*i *_in the three considered approaches, we see that the two procedures with constant specific activity (methods "b" and "c") exhibit a greater uncertainty on K_*i *_when compared to the constant activity procedure (method "a"). Indeed, in the former cases, components due to volume or mass reading must be added to the uncertainty on measurement reproducibility. Consequently we can expect a greater accuracy for the constant activity procedure in determining volume correction factors. Moreover, comparing expressions (10) and (12), it follows that uncertainty contributions due to the syringe volume reading could be significantly larger than those due to mass determinations, because of the improved accuracy of the analytical balance reading with respect to syringe graduation. By this preliminary theoretical analysis of the three considered procedures, we can conclude that, while the accuracy on K_*i *_in the constant activity mode is only affected by the reproducibility of the calibrator response, in the constant specific activity procedures the precision in the determination of the various radiopharmaceutical quantities inside the syringe is crucial for the method accuracy.

**Figure 1:**
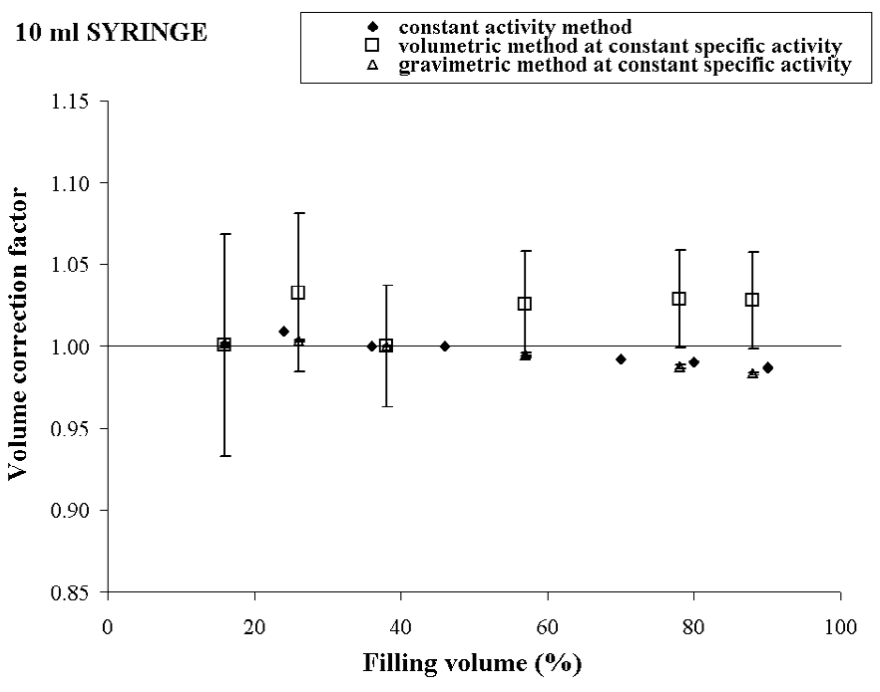
Experimental volume calibration curves and uncertainties for 10 ml syringe and three different procedures.

**Figure 2:**
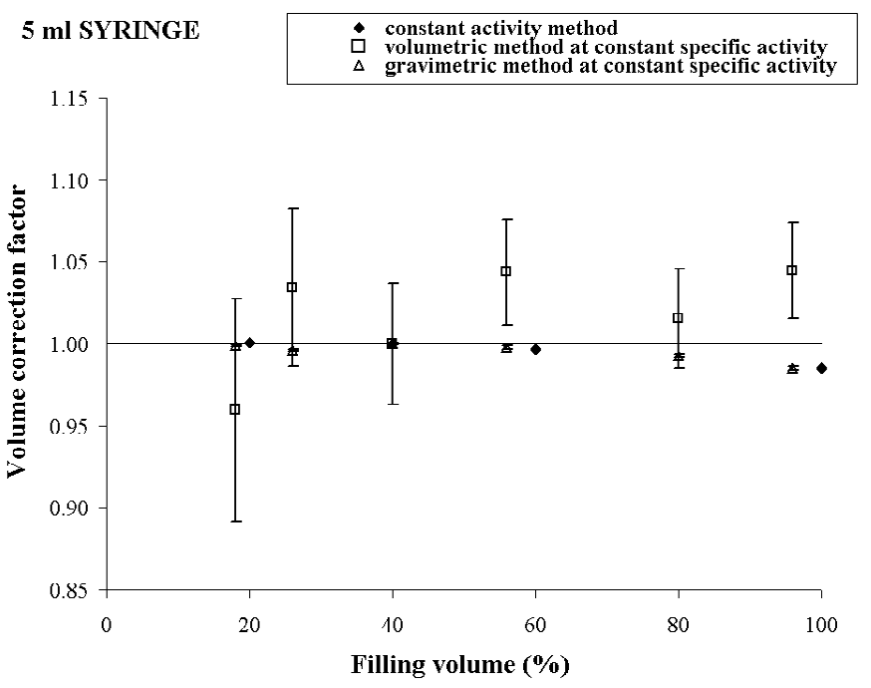
Experimental volume calibration curves and uncertainties for 5 ml syringe in three different procedures.

**Figure 3:**
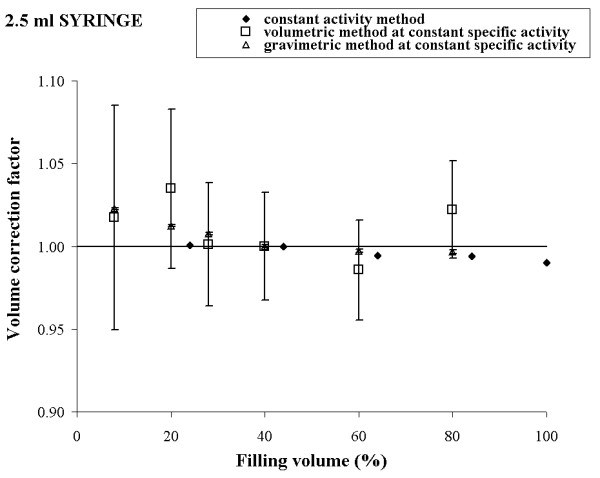
Experimental volume calibration curves and uncertainties for 2.5 ml syringe in three different procedures.

**Figure 4:**
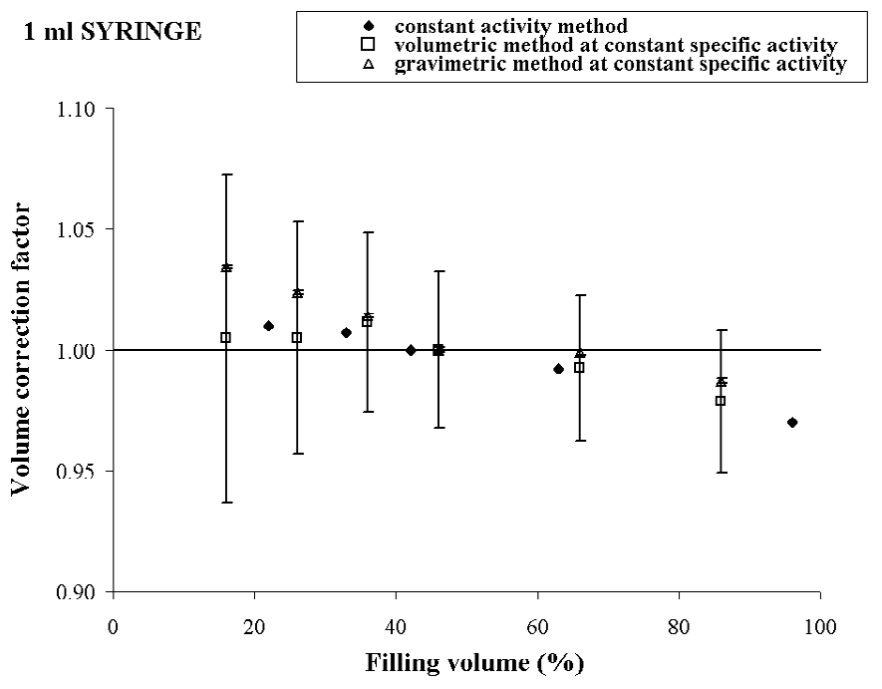
Experimental volume calibration curves and uncertainties for 1 ml syringe in three different procedures.

To check our theoretical conclusions, the three methods described above were experimentally implemented using a Tc-99m solution. The results obtained for 10 ml, 5 ml, 2.5 ml, and 1 ml syringe are reported in figures 1, 2, 3 and 4, respectively.

For each syringe, the plot shows a comparison of the volume calibration curves obtained by the three different procedures and the corresponding uncertainties calculated according to expressions (4), (10) and (12).

## Discussion

Observing the figures 1, 2, 3, 4, a good agreement between the values of K_*i *_obtained by the method a) and the method c) for all the considered syringes is evident. The percentage deviation of the experimental data was within 2 % for all the considered syringes except the 1 ml syringe, for which it was about 4%.

The overall uncertainty of the correction factor is always negligible in the method a) while it varied from about 0.1 % (10 ml syringe) to 0.5 % (1 ml syringe) in the method c).

In method b), the behaviour of the experimental data was quite irregular for each type of syringe and the percentage deviation reached 10 % (5 ml syringe).

The overall uncertainty on the correction factor was always rather high and it varied from 10 % (10 ml syringe) to 25 % (5 ml syringe).

## Conclusion

In this paper we reported a comparison between three experimental methods to derive volume correction factors for a PET DOSE activity calibrator, used in our Institution.

The agreement between the experimental data obtained in the constant activity method and gravimetric method at constant specific activity and the small associated uncertainties show the accuracy of these two procedures. On the contrary, the large uncertainties on volume reading obtained by the volumetric method at constant specific activity could lead to a wrong evaluation of the correction factors.

The agreement between the first two approaches should also be tested for low energy beta emitting radionuclides, like ^90^Y [5,6], ^111^In and ^18^F [7], where measurement geometries and filling volume could affect the calibrator linearity and volume correction factor value.

## Competing interests

The authors declare that they have no competing interests.

## Authors' contributions

^§ ^Corresponding author.

*These authors contributed equally to this work.
